# Investigation into Host Selection of the Cecal Acetogen Population in Rabbits after Weaning

**DOI:** 10.1371/journal.pone.0158768

**Published:** 2016-07-05

**Authors:** Chunlei Yang, Lan Mi, Xialu Hu, Jianxin Liu, Jiakun Wang

**Affiliations:** Institute of Dairy Science, MoE Key Laboratory of Molecular Animal Nutrition, College of Animal Sciences, Zhejiang University, Hangzhou 310058, China; MJP Rohilkhand University, INDIA

## Abstract

Homoacetogenic bacteria have received attention as a hydrogenotrophic population that offers a significant energetic advantage to the host animal. Reductive acetogenesis is likely an important hydrogen disposal mechanism in the cecum of rabbits. However, molecular ecology information about cecal acetogen candidates has rarely been reported. To better understand the effect of host selection in the rabbit cecal acetogen community with respect to growth, rabbits at four different age stages (30, 60, 120 and 180 days) with the same diet were studied. Although the abundance of potential acetogens and methanogens was high in the cecum of rabbits undergoing growth, many novel potential acetogen populations were observed in the cecum of rabbits across all age groups. Young and adult rabbits had their own distinct acetogen community although they received the same diet, which suggests that as the rabbit ages, acetogens in the cecum undergo developmental changes because of host selection that are independent of diet, and perhaps the different acetogen communities result in different hydrogenotrophic characteristics. The within-group similarity increased with age, indicating that the acetogen community converges to a more homogeneous and stable arrangement with aging.

## Introduction

Homoacetogenic bacteria (acetogens) are a group of obligate anaerobic bacteria that utilize the acetyl coenzyme-A (CoA) pathway to synthesize acetate from syngas [1]. Acetogenesis is of great importance to the global carbon cycle. Approximately 10^13^ kg of acetate is formed annually in anaerobic habitats [2]. Acetogens are phylogenetically quite diverse and metabolically versatile [3, 4], but only some strains, such as *Moorella thermoacetica* [5, 6], *Clostridium ljungdahlii* [7, 8], *Clostridium ragsdalei* [8, 9] and their genetic modifications, are used in syngas fermentation for biofuels. Furthermore, homoacetogenesis is a promising pathway to compete with methanogens in the rumen, because methane producers cause 23% of the global anthropogenic methane emissions [10]. A better understanding of the molecular ecology of the acetogen population will help develop new acetogen products and enhance its catalyst function.

Methanogens have a greater thermodynamic advantage than acetogens when competing for hydrogen in anaerobic habitats [11–13]. Rabbits, as herbivores, have less methane production [14] and lower energy loss from methane production per unit of body mass than ruminants [15]. The estimated hydrogen recoveries for methane are 24.7% and 85.4% in the rabbit cecum [16] and goat rumen [17], respectively, and the ratio of acetate to propionate production is much higher in the rabbit cecum compared to rumens (17.8 vs. 2.81) [18]. The reason for this may be because reductive acetogenesis is the dominant hydrogen disposal pathway in the cecum of rabbits [19, 20]. Therefore, acetogens that are, more efficient at syngas fermentation may exist in the cecum of rabbits.

The structure and composition of the gut microbiota is driven by lifestyle strategies, such as the growth rate, substrate utilization patterns, and host selection for specific bacteria with emergent collective behavior that is beneficial to the host [21]. Studies on the bacterial 16S rRNA gene revealed that the fecal microbiota of mammals is specific and rather stable for their particular host species, to a large extent [22–24], suggesting that mechanisms exist to recruit and maintain selected bacterial populations.

The cellulolytic capability of the gut microbiota is enhanced with an increase in the growth stage of the host [25, 26], and hence, more hydrogen is produced. However, there is a lack of knowledge on the stability or fluctuations in the cecum of rabbits maintained on a uniform diet and the selection of the acetogen population under this stability or fluctuation. Knowledge of the acetogen distribution with growth stage will help us understand the microbial host selection process and develop new acetogen products in the future. Therefore, host selection of the cecal acetogen population was studied in the cecum of rabbits at different growth stages maintained under constant conditions, including a uniform diet.

## Materials and Methods

### Experimental design and sampling

The Animal Care Committee of Zhejiang University (Hangzhou, China) approved all experiments, and the experimental procedures used in this study were in accordance with the university’s guidelines for animal research. Rabbits at four different age stages were fed the same diets and were used to study the change in the acetogen population in the cecum. Six male New Zealand White rabbits at ages 30, 60, 120 and 180 days were purchased simultaneously from the Zhejiang Academy of Agricultural Science. They were housed in indoor three-layer cages (60×50×35 cm) with natural lighting and raised with the same commercial pellet diets consisting of 12% corn, 18% bran, 8% soya bean cake, 31% meal, 10% malt root, 16% chaff, 0.8% powder, 0.5% salt and 4% commercial additives. Only one rabbit was housed in each individual cage. All rabbits were sacrificed on the same day. The performance of euthanasia and removal of cecum were undertaken by trained animal technician (license number: 15128, issued by Zhejiang University Laboratory Animal Center). Body weight was measured for each rabbit to ensure intravenous injection of 100 mg/kg (body weight) phenobarbital sodium (Sigma, Saint Louis, MO, USA) [27]. Once the rabbits have lost toe pinch, leg withdrawal reflex, ear intravenous injection of 20 ml air [28] was then performed to accelerate the death of rabbit. After sacrifice, the cecum was immediately removed and isolated by tying off the extremities with a nylon string to prevent movement of the digesta. The contents of the proximal, medial and distal parts of the cecum were squeezed into 50-ml sterilized eppendorf tubes and mixed for pH measurement. The samples were stored on dry ice before shipment to laboratory where they were then stored at -80°C.

### Measurement of cecal fermentation parameters

The samples for the measurement of volatile fatty acids (VFA) and ammonia nitrate were prepared as described by Yang et al. [29]. Approximately two grams of each stored sample was vortexed with 9 ml sterile PBS (pH 7.0). Then the supernatant was obtained after centrifugation at 13,000 × g at 4°C for 15 min. Colorimetry [30] and gas liquid chromatography (GC-2010, Shimadzu, Kyoto, Japan) [31] were used to determine the ammonia nitrate and VFA concentrations, respectively.

### DNA extraction and real-time PCR quantification

Total DNA was extracted from 0.1 gram of each of the stored samples using the cetyltrimethylammonium bromide (CTAB) method as described by Gagen et al. [32]. Primers (Table 1) specific for the formyltetrahydrofolate synthetase gene (*fhs*) [33] and the methyl coenzyme-M reductase A gene (*mcrA*) [34] were used to amplify potential acetogens and methanogens, respectively. The 16S rRNA gene from total bacteria was amplified with primers as reported by Denman and McSweeney [35]. Real-time PCR was performed with SYBR green in an ABI 7500 (Life Technologies, Singapore) using the following program: one cycle of initial denaturation at 95°C for 10 s, followed by 40 cycles of denaturation at 95°C for 15 s and annealing at 60°C for 1 min. The standards were prepared from serial dilutions of the linearized plasmid containing between 10^2^ and 10^9^ target gene copies calculated from the concentration of plasmids. The copy numbers of the standards and the copy numbers of the target genes per gram of cecal sample were calculated according to the method of Ritalahti et al. [36].

**Table 1:** Primers used in this study.

Target gene	Forward primer	Reverse primer	Size (bp)
16S rRNA gene of TB	CGGCAACGAGCGCAACCC	CCATTGTAGCACGTGTGTAGCC	130
*fhs*	GTWTGGGCWAARGGYGGMGAAGG	GTATTGDGTYTTRGCCATACA	250
*acsB*	CTBTGYGGDGCIGTIWSMTGG	AARCAWCCRCADGADGTCATIGG	216
*mcrA*	TTCGGTGGATCDCARAGRGC	GBARGTCGWAWCCGTAGAATCC	140

*fhs*: formyltetrahydrofolate synthetase; *acsB*: subunit B of acetyl-CoA synthase; *mcrA*: methyl coenzyme-M reductase A; TB: total bacteria.

### Analysis of the acetogen community

Amplification of subunit B of the acetyl-CoA synthasegene (*acsB*) from each extracted DNA sample was performed using the TaKaRa PCR Kit (TaKaRa, Dalian, China) with 1.0 ng template DNA per reaction. The specific primers for *acsB* [32] (Table 1) were used with the addition of 6-base barcodes and an Illumina adapter sequence [37]. Amplification was performed on the ABI 9700 PCR System using the conditions described by Gagen et al. [32]. The PCR products for each sample were separated from the primers and primer dimers by electrophoresis on a 1.5% agarose gel and the QIAquick gel extraction kit (QIAGEN, Shanghai, China). Then, the products for each sample were mixed in equal ng quantities using Qubit 2.0 (Invitrogen, USA) and sent to a commercial company (Novogene, Beijing, China) for Illumina MiSeq sequencing.

After sequencing, the size filtering, quality control (Q>20) and chimera removal of the raw data were performed using the pipeline in QIIME [38]. The sequences that passed quality control were aligned in Mega version 4.0 [39] to strictly select the sequences that were approximately 216 bp long and contained the forward and reverse *acsB* primers. Then, these nucleotide sequences were translated into amino acid sequences in a specific open reading frame using the translate tool in ExPASy [40]. The obtained ACS amino acid sequences were aligned again and length-based filtering was performed. The final obtained ACS amino acid sequences were clustered into operational taxonomic units (OTUs) at 0.05 distance limit using the CD-HIT program [41]. Additional analysis of OTUs was performed in the R packages phyloseq, DEseq, ggplot2 and gplots [42–45]. RAxML version 7.0.3 [46] and ARB [47] were used to construct the maximum-likelihood trees of the deduced ACS amino acids as described by Gagen et al. [32]. The sequences obtained in this study were deposited in the European Nucleotide Archive (ENA) under accession number PRJEB12535.

### Statistical analysis

The abundance of the 16S rRNA and *fhs* and *mcrA* genes, and the concentration or molar percentage of the total and individual VFA were analyzed by one-way analysis of variance in R 3.2 with individual rabbits as the experimental unit and age as the main effect. Multiple comparisons of means among different age groups were conducted using Turkey multiple range tests. Significance was declared at P<0.05.

## Results and Discussion

### Change in the cecum environment with growth stage

The pH of the cecum increased with age. However, the total VFA and ammonia nitrate decreased with age (Table 2). The ratio of acetate to propionate did not change significantly but was higher than 9.81:1. The primary activity of the bacterial population that resides in the hindgut is fermentation, and the major products are VFA. These can be absorbed across the gut mucosa and utilized by the rabbit as a significant energy resource [48]. With increased body weight, the energy requirement should also increase; therefore, the decrease in the total VFA in the cecum of older rabbits may be a result of increased absorption. The high ratio of acetate to propionate further confirms that acetogenesis is an important metabolic pathway present in the rabbit cecum.

**Table 2:** Fermentation parameters of cecal content.

Item	30 d	60 d	120 d	180 d	SEM	P values
pH	—	6.31^b^	6.42^b^	6.71^a^	0.08	<0.01
Ammonia nitrate (mg/g)	5.32^a^	5.34^a^	3.34^b^	1.50^c^	0.57	<0.01
Volatile fatty acids (mmol/g)	40.03^a^	34.86^a^^b^	33.22^b^	30.88^b^	1.82	0.04
Acetate (%)	74.46	78.42	77.42	75.86	1.51	0.37
Propionate (%)	7.14	6.85	6.99	7.45	0.29	0.65
Butyrate (%)	18.40	16.93	16.88	19.64	0.80	0.18
Acetate: Propionate (A: P)	10.59	11.17	11.14	9.81	0.71	0.40

^a-c^ Values with different superscripts in the same row differ significantly (*P*<0.05). The data are the means.

### Comparison of the acetogen community

**Fig 1:**
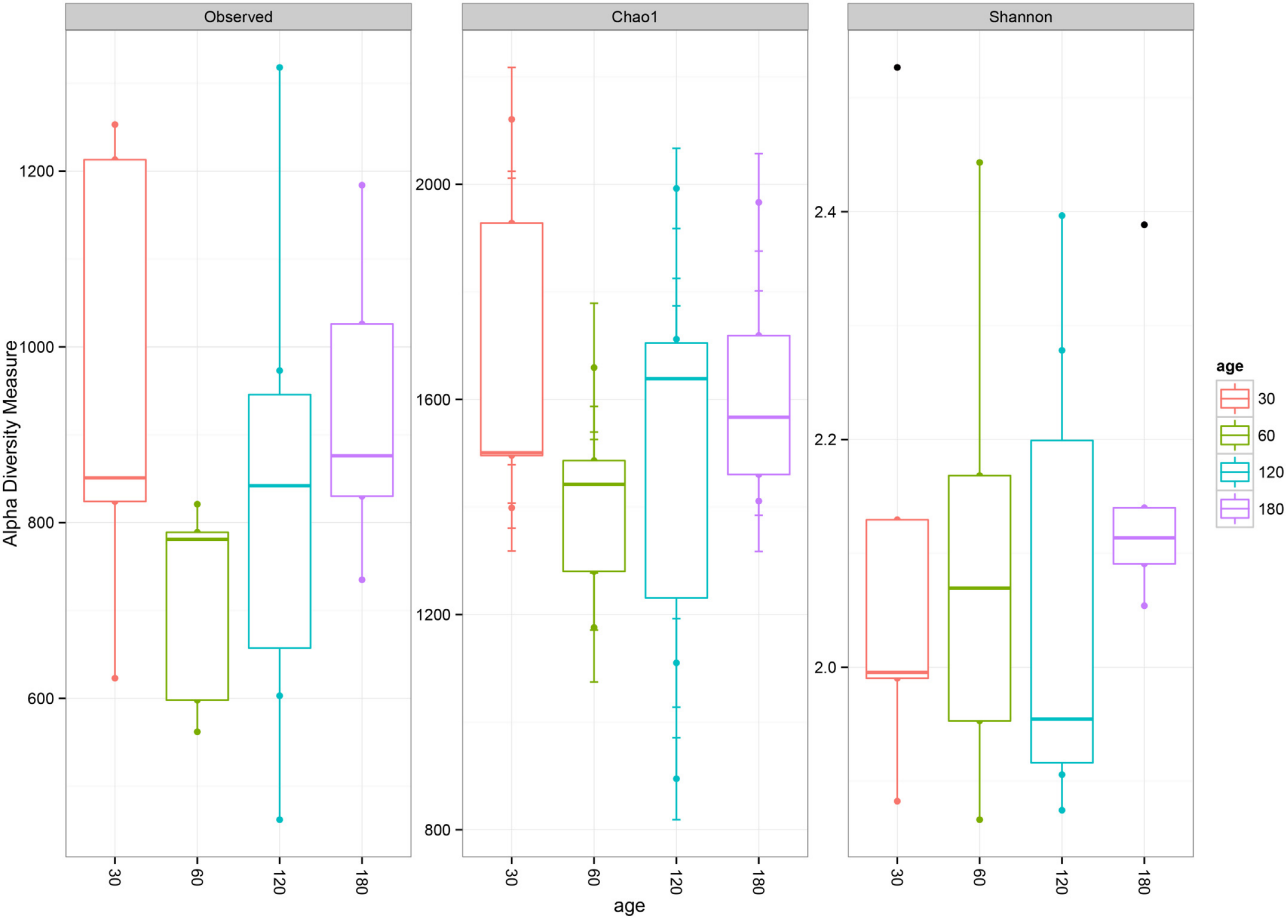
Alpha diversity measures of the deduced ACS amino acid sequences recovered from the cecum of rabbits across different age groups. Observed = Observed OTU numbers; Chao1 = Chao estimator; Shannon = Shannon diversity index.

The deduced amino acids for *acsB* from the cecum of rabbit clustered into 6101 OTUs, suggesting that many acetogen species exist in the cecum of rabbits. However, most of the detected sequences lack representatives from previously described sequences, which is in consistent with previous studies [32, 49], suggesting that the novelty of the rabbit cecal flora is likely because the rabbit is a cecotrophic species. In addition, these novel *acsB* sequences amplified in our study expand the database of ACS amino acid sequences for acetogens. Alpha diversity, which is described in terms of richness or the number of different species present, and evenness were initially investigated to observe changes in acetogen diversity in the cecum of rabbits during growth and development (Fig 1). Our results showed that both the richness and evenness of acetogen populations in the cecum of rabbit at each growth stage did not change significantly (P>0.05), with no significant changes in the Chao 1 and Shannon indices (Fig 1). However, young rabbits showed a larger animal variance compared to the older rabbits (Fig 1), which indicates that during the first days of life, the acetogen population in the cecum has more heterogeneity and becomes more homogeneous and stable with growth.

**Fig 2:**
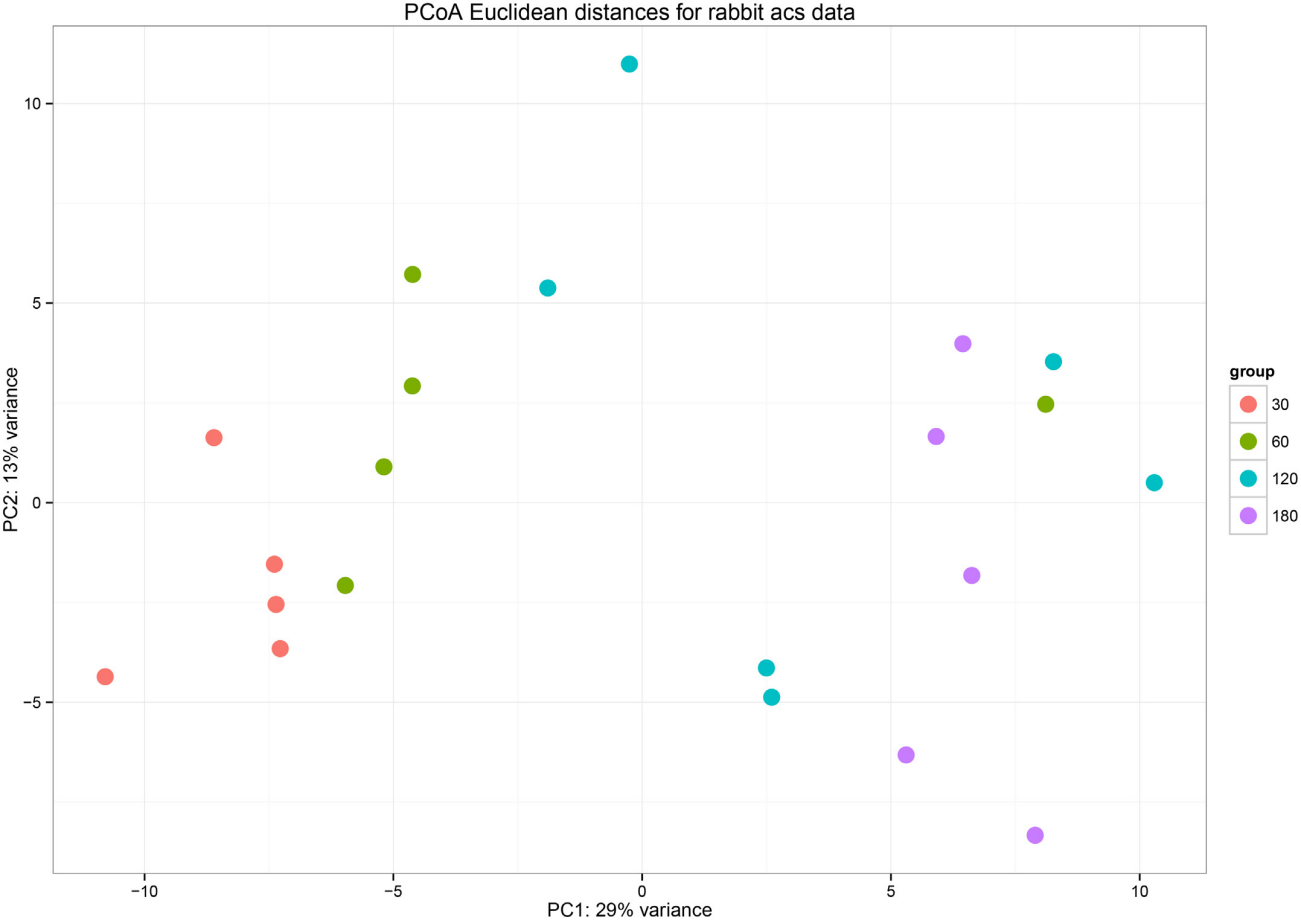
Covariation of the community structure of potential acetogens in the cecum of rabbits at different age stages using principal coordinate analysis (PCoA). The PCoA plot shows that the samples from different age groups span their first two principal components, principal coordinate 1 (29% of variance) and principal coordinate 2 (13% of variance).

The structure of the acetogen population was also analyzed with beta diversity measures using principal coordinates analysis (PCoA) (Fig 2). Clear alterations of the acetogen community in the cecum of rabbit were observed with growth. Animals at different growth stages were divergent in their acetogen composition, suggesting that rabbits at each growth stage have their own distinct acetogen community, although they received the same diet. Variations in the cecal acetogen community between animals at the same growth stage were also observed, suggesting that the effect of rabbit-specific host selection determines the distinct acetogen population in each animal.

**Fig 3:**
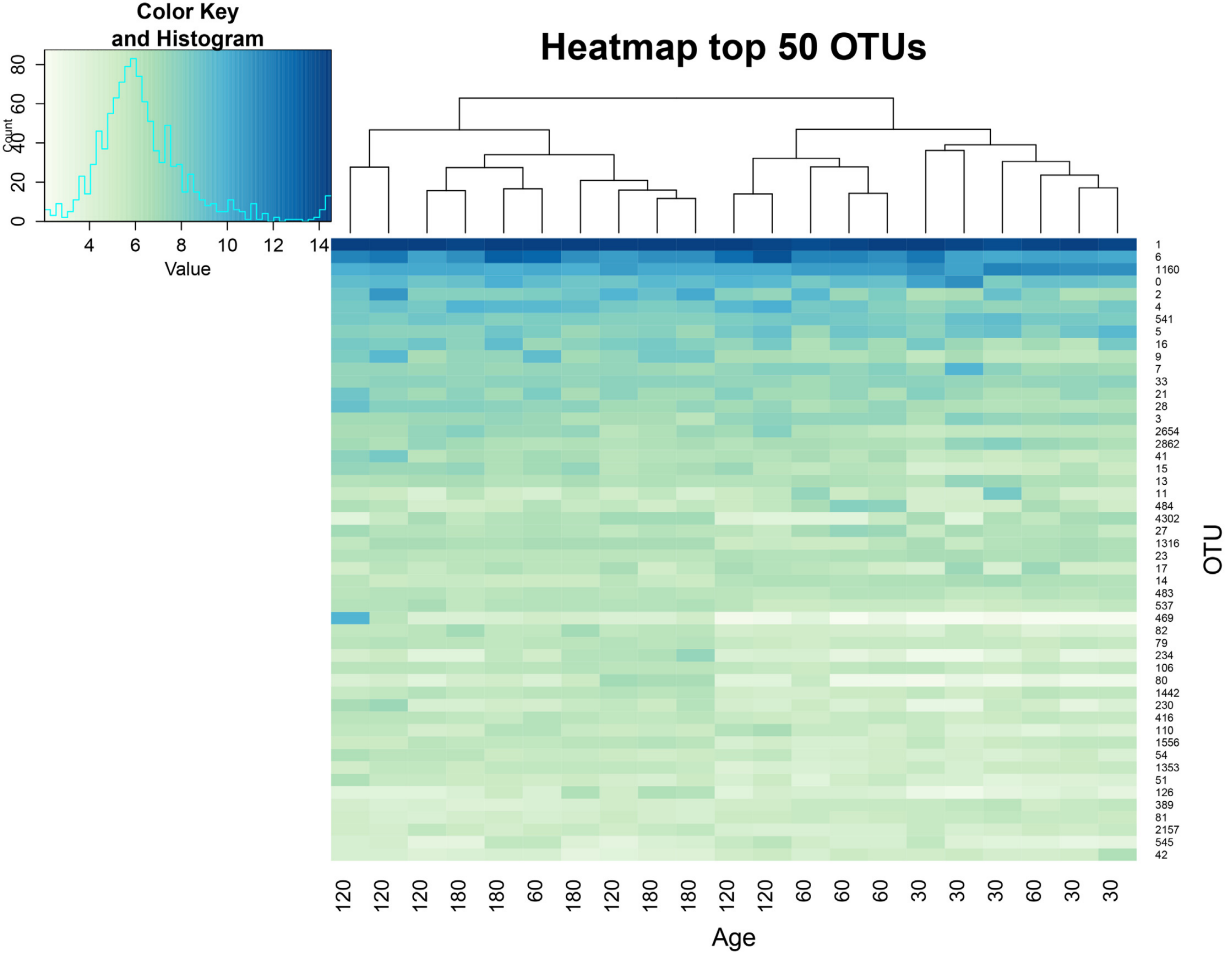
Assessment of differentially expressed ACS genes in samples across all age groups in heat map format. A heat map showing the rlog transformed expression data of the first 50 ACS amino acid sequences in the top ranked ACS sequences detected in the cecum of rabbits of different ages. The color scale ranges from blue (high expression) to white (low expression).

**Fig 4:**
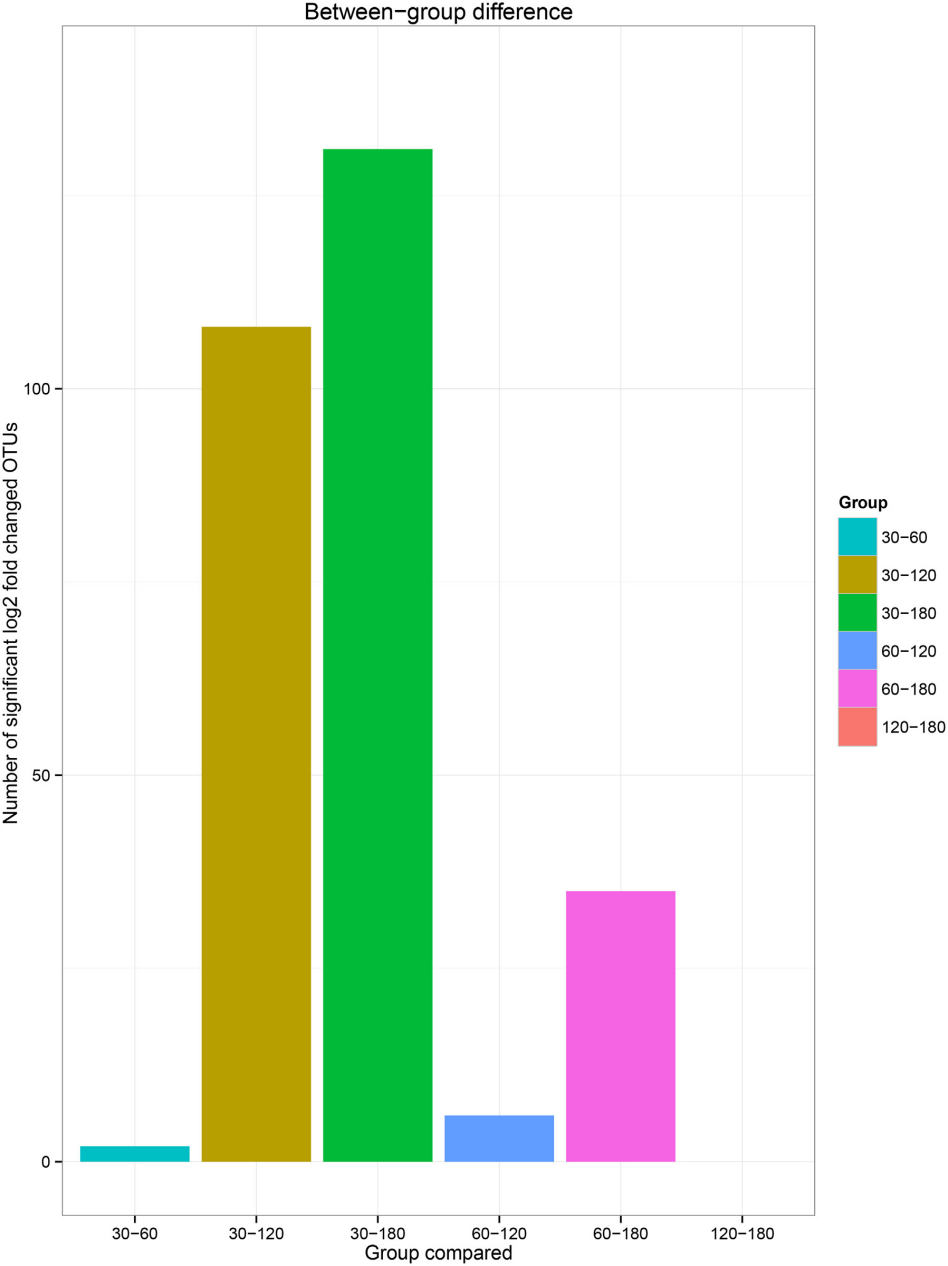
Analysis of the within-group similarity of two different age groups. The higher number of significantly changed expressed deduced ACS amino acid sequences between two age groups with a lower similarity between them.

The between-group similarity was further analyzed using a heat map (Fig 3), and the number of presented OTUs was significantly changed between groups at each of the two growth stages (Fig 4). Obvious differences were found in the presented ACS OTUs between the growth stage and host animals (Fig 3) by observing the clusters. The groups aged 30 and 60 days were clearly separated from the groups aged 120 and 180 days, with the exception of one rabbit at 60 days and two rabbits at 120 days. Furthermore, a significant individual effect was observed because rabbits of the same age showed different sub-clusters. From analysis of the number of OTUs that were significantly changed (fold change ≥2) (P<0.05) in every two age groups (Fig 4), the between-group similarity increased in a growth-dependent manner. There were 131 ACS OTUs that showed a greater than 2-fold change between groups aged 30 and 180 days, whereas the changed OTUs were reduced to 35 and 0 when the 180-day group was compared to 60 days and 120 days groups, respectively, suggesting that the cecum of rabbits becomes a more stable arrangement of the acetogen community from youth to adult. Similar results were observed from previous studies focusing on the bacterial community. The pairwise similarity of human bacterial diversity increases with age [50], and the gut microbiome also undergoes a convergence to a mature bacterial arrangement with age [51].

### Phylogenetic analysis of the acetogen community

**Fig 5:**
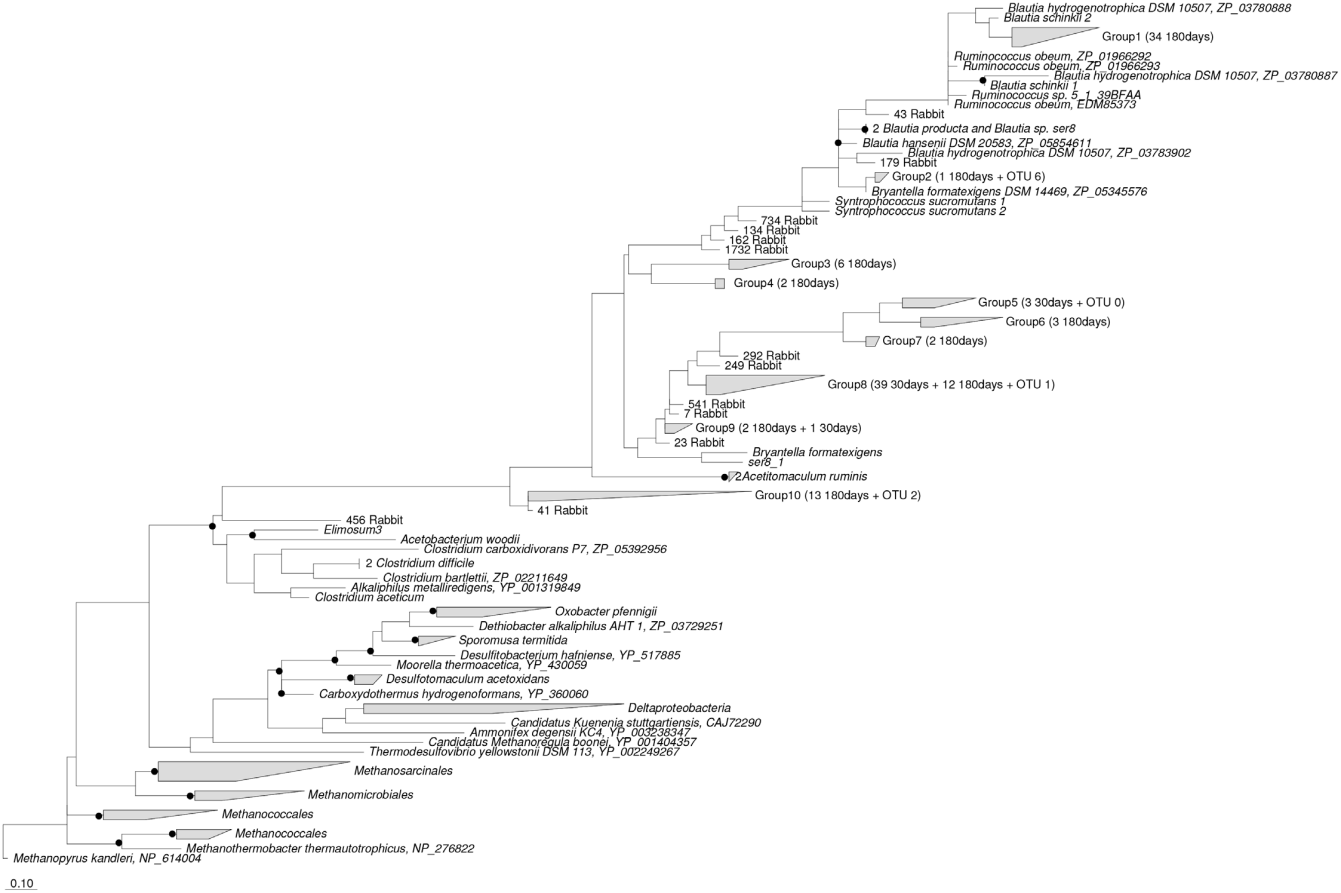
Phylogenetic analysis of significantly changed (fold change ≥2) acetyl-CoA synthase amino acid sequences in the cecum of rabbits aged 30 and 180 days with reference ACS sequences (GenBank accession numbers are shown after the species names). Bootstrap values that ≥75% are shown at the nodes. The scale bar represents a sequence divergence of 10%. The number of ACS OTUs more highly present in the 30-day group or the 180-day group, as well as the dominant OTUs across all age groups, is indicated in brackets.

Due to the limited *acsB* gene database, most of the detected novel ACS OTUs in the cecum of rabbits could not be classified at the species or genus level. Therefore, a maximum likelihood tree (Fig 5) was constructed to determine the phylogenetic information of acetogens at the family level. In a previous analysis, the groups of 30 and 180 days showed the largest between-group difference. To identify the potential link between acetogen species and young and adult growth stages, the presence of OTUs that changed significantly and the dominant OTUs that were not different in the 30- and 180-day groups were included in the maximum likelihood tree (Fig 5). The most abundant OTU, OTU 1, was affiliated with the *Lachnospiraceae* family but was phylogenetically placed far away from any of the known isolates present across all growth stages. OTU 1 accounted for 57.66% of all species detected in our study, showing an unchanged abundance between the 30- and 180-day groups; this was followed by OTU 6 with a proportion of 10.73%, and it was phylogenetically affiliated with the *Blautia* group (Fig 5). The presence of 85 ACS OTUs (a total of 61 in groups 1, 2, 3, 4, 6, 7 and 10; 12 in group 8; 2 in group 9; as well as OTUs 41, 43, 79, 134, 162, 249, 292, 456, 734, and 1732 rabbit) significantly increased (fold change ≥2) (P<0.05), and 46 ACS OTUs (3 in group 5; 39 in group 8; 1 in group 9; and OTUs 7, 23, and 541 rabbit) significantly decreased (fold change ≥2) (P<0.05) in the adult rabbit aged 180 days (Fig 5). These significantly changed (fold change ≥2) ACS sequences were phylogenetically placed at 23 different phylogenetic places in the maximum-likelihood trees of ACS amino acid sequences (Fig 5). Comparison of the tree topology between the 16S rRNA gene and the ACS amino acid sequences published previously show [32] the presence of a total of 34 ACS OTUs (group 1), including a dominant OTU, OTU 4, which clustered together in the ACS tree placed phylogenetically between the *Blautia* group and *Ruminococcaceae*, as well as one OTU in group 2 and OTU 179, which was phylogenetically affiliated with the *Blautia* group, that significantly (P<0.05) increased in adult rabbits aged 180 days compared to the young rabbits aged 30 days. The presence of OTU 43, which was phylogenetically affiliated with *Ruminococcaceae*, significantly (P<0.05) increased in older rabbits aged 180 days. In addition, another 13 ACS OTUs (group 10) and OTUs 41 and 456 were phylogenetically affiliated with *Clostridiaceae*, showing a significant increase (P<0.05) in rabbits aged 180 days. Another 8 clusters (groups 3, 4, 6, and 7 and OTUs 41, 134, 162, 249, 292, 456, 734, and 1732) of ACS sequences containing the dominant OTU 9 were phylogenetically affiliated with the *Lachnospiraceae* family and were more highly present (P<0.05) in rabbits aged 180 days but were far away from any known cultured acetogens. The presence of OTUs 7, 23, and 541 and group 5 in the trees was affiliated with *Lachnospiraceae* but were far away from any known cultured acetogens and was higher (P<0.05) in young rabbits aged 30 days. A previous study indicated that over 90% of the bacterial sequences in the cecum of rabbit belong to the *Firmicutes* phylum [52]. *Ruminococcaceae* and *Lachnospiraceae* were the dominant families, accounting for 45% and 35% of all sequences, respectively [53]. In our study, the great diversity of ACS sequences was broadly placed phylogenetically into the family groups *Lachnospiraceae*, *Ruminococcaceae*, *Blautia*, and *Clostridiaceae*, which were shown from the maximum-likelihood trees of the 16S rRNA gene and the ACS amino acid sequences for comparison to the tree topology reported by Gagen et al. [32]. Adult rabbits at 180 days showed a more diverse phylogenetic acetogen community compared to young rabbits aged 30 days. These phylogenetic differences in the potential acetogen populations in the cecum of young and adult rabbits may contribute to their different roles in hydrogenotrophy and energy utilization in the cecal ecosystem. Previous studies showed that a novel sequence, closely related to the known reductive acetogen *Blautia coccoides*, was associated with carbon dioxide and hydrogen metabolism [54]. Mixotrophic acetogens, which can simultaneously use hydrogen and organic substrates for growth, may be more energetically competitive with methanogens in some situations [55].

### Comparison of bacteria and archaea populations

**Fig 6:**
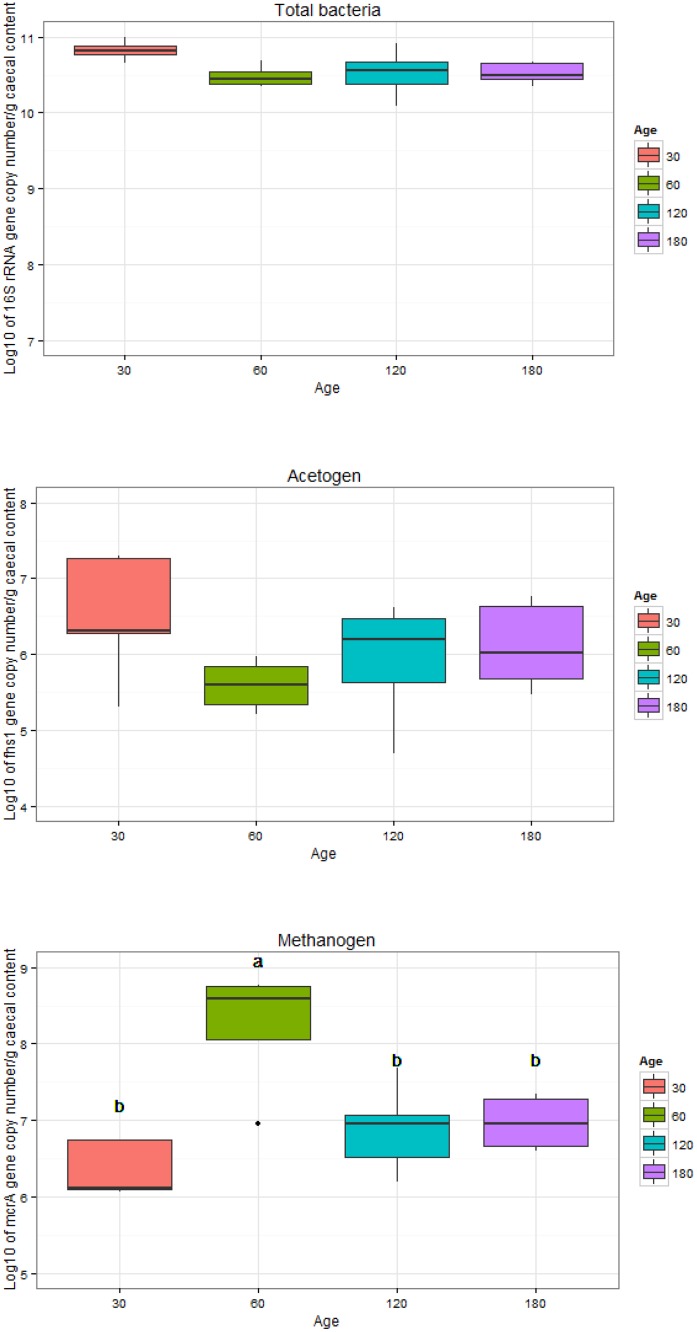
Abundance of the *fhs*, *mcrA*, and 16S rRNA genes in total bacteria in samples from different age groups. Groups with different superscripts indicate that they differ significantly (P<0.05).

From analysis of the gene copy number of the 16S rRNA targeting total bacteria, large amounts of bacteria were observed in the cecum of rabbits across all growth stages (P>0.05) (Fig 6), which is consistent with previous studies [56, 57]. Real-time PCR was performed to specifically quantify the gene copy number of *fhs* and *mcrA* to determine the potential abundance of acetogens, which is currently the best target for quantifying acetogens [33, 58], and with the *mcrA* gene to determine the methane producing hydrogenotrophy, which is likely the dominant hydrogen disposal method in most anaerobic environments. The abundance of the *mcrA* gene was at 10^6^ to 10^8^ gene copy numbers per gram of cecal content in most of the samples, which is consistent with a previous study (approximately 7.4 log_10_ copy number per gram of cecal content at day 35) [52] and was significantly higher (P<0.05) in samples aged 60 days compared with the other age groups (Fig 6). However, the abundance of the *fhs* gene was at 10^5^ to 10^6^ gene copy numbers per gram of cecal content in most of the samples and showed no significant differences (P>0.05) with growth stage (Fig 6).

## General Discussion

Compared to the published acetogen strains used in microbial production platforms, many new acetogen strains were found in the cecum of rabbits in our study, with the dominant acetogen affiliated with the *Blautia* family, in which the known strains *Blautia coccoides*, *Blautia hydrogenotrophica* and *Blautia schinkii* can use carbon dioxide and hydrogen to synthesize acetate as a main product [59, 60]. In addition, the minimum doubling time of *Blautia producta* with carbon monoxide or carbon dioxide and hydrogen as a substrate is only 1.5 or 5 h [61, 62], suggesting that the cecum of the rabbit is a promising resource for harvesting novel acetogen strains with potential industrial applications.

The true extent of acetogen biodiversity in the cecum of rabbits is not known because most organisms cannot be cultured ex vivo. However, there was a clear rabbit-specific effect on the cecal acetogen community in individuals used in this study. Only rabbits of the same species (New Zealand White rabbit) and with the same type of diet were used, which minimized both the genetic variability and dietary variation in the study to examine the effect of growth. However, because of the limited database of known acetogens, the acetogen strain changes with growth could not be classified accurately. Some of the acetogen strains were more present in adult rabbits (180 days) and were affiliated with the *Blautia* group, *Ruminococcaceae* and *Clostridiaceae*. *Blautia schinkii* and *Blautia hydrogenotrophica* are known to use carbon dioxide and hydrogen as substrates for growth, and *Blautia* spp. may play a key role in the kangaroo foregut and are associated with carbon dioxide and hydrogen metabolism [54]. The *Clostridiaceae* family contains the *Clostridium* genus, which is now an important candidate in the biofuel industry. Therefore, because of the growth effect from host selection, the cecum of adult rabbits may be a good source for enriching acetogen candidates for biofuel production.

The inter-individual variation of the cecal bacteria in rabbits was previously observed, although acetogen was not specifically tested [52]. Comparable results were also described regarding the bacterial community in the feces of ponies. A significantly different population was found in each pony during 11 weeks of observation with the same diet [63]. In addition, from the analysis of the bacterial community in cattle and human, the diversity and within-group similarity increases with growth, suggesting a convergence to a more diverse, but homogeneous and specific, mature bacterial arrangement with growth [25, 51].

As a hindgut fermenter, the greatest numbers of bacteria reside in the cecum of rabbits. Relationships between the gut bacterial communities and their mammalian hosts contribute to the host’s well-being and proper function [22, 64]. In the cecum, archaea and acetogens play important roles in the final stage of organic matter decay, reducing carbon dioxide into methane or acetate, and the latter plays an important role in host animal nutrition [65]. Although high levels of gene copy number both for *mcrA* and *fhs* were observed in our study, a previous study indicated that the cecum of rabbits harbors a methanogenic community with low complexity of the genus *Methanobrevibacter*, and it is possibly one-species dominated because all sequences detected presented similarity values of 99% or higher [66]. However, many novel and host-specific acetogen populations were observed in our study, suggesting an important role for reductive acetogenesis in the cecum of rabbits.

To better understand the function of acetogens in the cecum of rabbit, further studies, such as tracking of the biological fate of carbon dioxide in the cecum of rabbits using RNA stable isotope probing (RNA-SIP) techniques, should be performed to identify the dominant organisms and pathways involved in hydrogenotrophy of rabbit cecum. This was previously performed in the kangaroo forestomach to explain the dominance of reductive acetogenesis as a hydrogen removal pathway [54].

## Conclusion

The cecum of the rabbit is a good resource to identify and develop new acetogen products. Hosts select preferable microbiota for their growth, and the microbiota in the cecum are more homogeneous and stable with growth and development. To better understand the contribution of acetogen populations to the host, stable isotope tracing can be performed to identify the dominant organisms and pathways involved in hydrogenotrophy in the cecum of rabbit.
